# *Fusarium* Fungi Produce Nitrous Oxide (N_2_O) from Nitrite (NO_2_^–^) in a Model Pot System Simulating the Soybean Rhizosphere

**DOI:** 10.1264/jsme2.ME24092

**Published:** 2025-05-01

**Authors:** Makoto Moriuchi, Keiichi Kuzunuki, Fumio Ikenishi, Reiko Sameshima, Akira Nakagiri, Sakae Toyoda, Chie Katsuyama, Kaori Kakizaki, Manabu Itakura, Naohiro Yoshida, Yuichi Suwa, Kiwamu Minamisawa

**Affiliations:** 1 Graduate School of Life Sciences, Tohoku University, Sendai 980–8577, Japan; 2 Department of Chemical Science and Engineering, Institute of Science Tokyo, Yokohama 226–8501, Japan; 3 College of Agriculture, Academic Institute, Shizuoka University, Shizuoka 422–8529, Japan; 4 Biotechnology Division, Tohoku Regional Office, National Institute of Technology and Evaluation, Sendai 983–0833, Japan; 5 Department of Biological Sciences, Faculty of Science and Engineering, Chuo University, Tokyo 112–8551, Japan; 6 Earth-Life Science Institute, Institute of Science Tokyo, Tokyo 152–8550, Japan; 7 National Institute of Information and Communications Technology, Koganei 184–8795, Japan

**Keywords:** nitrous oxide, soybean nodule, *Fusarium*, site preference, stable isotope

## Abstract

Nitrous oxide (N_2_O) is a key atmospheric greenhouse gas that contributes to global warming, with anthropogenic N_2_O emissions from agriculture being a particular concern. Among agricultural sources, unknown soil organisms in the legume rhizosphere emit N_2_O from degraded root nodules. To discriminate between fungal and bacterial N_2_O emissions, we adopted an isotopomer ana­lysis, which provides site preference values (the difference in ^15^N abundance of the central and terminal N atoms in the N_2_O molecule). The addition of nitrite instead of nitrate to soybean nodulated roots significantly increased SP_N2O_ from –3.5‰ to 4.2‰ in a pot system. Moreover, a mutation of the *nirK* gene (encoding dissimilatory nitrite reductase) in symbiotic bradyrhizobia significantly increased SP_N2O_ from 4.2‰ to 13.9‰ with nitrite. These results suggest that nitrite-utilizing N_2_O emissions via fungal denitrification occurred in the model pot system of the soybean rhizosphere. Microscopic observations showed fungal hyphae and crescent spores around N_2_O-emitting nodules. Therefore, we isolated single spores from soybean nodules under a microscope. A phylogenetic ana­lysis revealed that all 12 fungal isolates were *Fusarium* species, which exist in soybean field soil. When these isolates were cultivated in glycerol-peptone medium supplemented with nitrate or nitrite (1‍ ‍mM), 11 of the 12 isolates strongly converted nitrite to N_2_O; however, no N_2_O emissions were noted in the presence of nitrate. A ^15^N-nitrite tracer experiment revealed that one N_2_O molecule was derived exclusively from two molecules of nitrite (NO_2_^–^) in the fungal culture. These results suggest that nitrite-utilizing *Fusarium* fungi mediate N_2_O emissions in the soybean rhizosphere.

The term *rhizosphere* was coined in 1904 by Lorenz Hiltner, who was interested in nitrogen cycles in the rhizosphere of nodulated roots of leguminous crops (Hartmann *et al.*, 2008). However, few studies have exami­ned microbial nitrogen transformations in the legume rhizosphere (Kuypers *et al.*, 2018; Sánchez and Minamisawa, 2019). One of the products of these transformations is the gas nitrous oxide (N_2_O), which, in addition to increasing global warming, causes stratospheric ozone depletion (Ravishankara *et al.*, 2009; Tian *et al.*, 2020). Terrestrial agricultural systems are the main anthropogenic sources of N_2_O (Tian *et al.*, 2020). Due to anthropogenic N_2_O emissions, the concentration of atmospheric N_2_O has increased by more than 20% from 270‍ ‍ppb in 1750 to 331‍ ‍ppb in 2018.

The soybean rhizosphere is a site of active N transformations, including the production and consumption of N_2_O (Sánchez and Minamisawa, 2019; Minamisawa, 2022). Field studies show that the emission of N_2_O starts during the late plant growth period (Inaba *et al.*, 2009). A ^15^N tracer experiment revealed that N_2_O emitted from the soybean rhizosphere was almost entirely derived from N_2_ that had been symbiotically fixed in the nodules (Inaba *et al.*, 2012). During nodule decomposition, organic N from nodules is mineralized into ammonium (NH_3_). N_2_O is then produced via nitrification and denitrification (Inaba *et al.*, 2009, 2012) (Fig. 1A).

Measurements of N_2_O flux from decomposed nodules formed with the wild-type strain and mutants of *Bradyrhizobium diazoefficiens* showed that this species played a role in N_2_O emissions via denitrification (41% of all N_2_O produced) (Fig. 1A) (Inaba *et al.*, 2012). Other denitrifying microorganisms in the soil also make important contributions, accounting for the remaining 59% of total N_2_O produced (Inaba *et al.*, 2012); however, these microbes have yet to be identified (Fig. 1A). In contrast, *B. diazoefficiens* strains carrying the *nosZ* gene exclusively reduce N_2_O, acting as a N_2_O sink (Inaba *et al.*, 2012). Net N_2_O flux in the soybean rhizosphere is assessed by the balance between N_2_O sources and sinks (Fig. 1A).

A useful tool to evaluate the contribution of each N_2_O production pathway in the environment is the N_2_O isotopomer ratio, which indicates the natural abundance of N_2_O isotopomers (^14^N^15^N^16^O and ^15^N^14^N^16^O) relative to that of ^14^N^14^N^16^O (Yoshida and Toyoda, 2000; Toyoda *et al.*, 2017). Differences in delta values between central N (N^α^) and terminal N (N^β^) nitrogen atoms, expressed as the metric site preference (SP=δ^15^N^α^–δ^15^N^β^), have specific values for each production pathway. N_2_O produced by bacteria has a SP value near 0‰, whereas fungal denitrification results in a SP value ranging from 16 to 37‰. (Toyoda *et al.*, 2005; Maeda *et al.*, 2015). We herein calculated SP values to identify the microbes relevant to N_2_O emissions from the soybean rhizosphere in a model pot system.

The aims of the present study were to identify and confirm unknown soil organisms other than bradyrhizobial endosymbionts that were relevant to N_2_O emissions in the model pot system simulating the soybean rhizosphere (Fig. 2).

Inaba *et al.* (2012) suggested that unknown soil microbes emit large amounts of N_2_O in the soybean rhizosphere, similar to soybean bradyrhizobia in our model pot system (Fig. 2). To identify some of these unknown soil microbes, we adopted a strategy combining the use of denitrification mutants of *B. diazoefficiens* USDA110 and a N_2_O isotopomer ana­lysis in the model pot system (Fig. 2). To eliminate N_2_O reduction, we used a *nosZ* minus strain (USDA110Δ*nosZ*) as a bradyrhizobial inoculant. To further eliminate N_2_O production by *B. diazoefficiens* USDA110, we used USDA110Δ*nirK*Δ*nosZ* defective in both the *nirK* and *nosZ* genes (Fig. 2B). In addition to denitrification mutants of USDA110Δ*nosZ* and USDA110Δ*nirK*Δ*nosZ*, we changed the inorganic nitrogen source from nitrate to nitrite in the model pot system (Fig. 1B).

## Materials and Methods

### Bacterial strains and media

The bacterial strains used in the present study were two denitrification mutants of *B. diazoefficiens* USDA110: USDA110Δ*nosZ* and USDA110Δ*nirK*Δ*nosZ* (Inaba *et al.*, 2012). *B. diazoefficiens* cells were grown at 30°C in HM salt medium (Cole and Elkan, 1973) supplemented with 0.1% arabinose and 0.025% (w/v) yeast extract (Difco).

### Model pot system

We previously developed a model system that imitates the field rhizosphere of nodulated soybeans during the late growth period (Inaba *et al.*, 2009, 2012). In the present study, we used this model system for the N_2_O isotopomer ana­lysis, fungal isolation, and the community ana­lysis (Fig. 2). Surface-sterilized soybean seeds (*Glycine max* cv. Enrei) were germinated in sterile vermiculite at 25°C for 2 days.

Each seedling was then transplanted into a Leonard jar pot (one plant per pot), which contained sterile vermiculite and nitrogen-free nutrient solution (Inaba *et al.*, 2012). Seedlings were inoculated with mutants of *B. diazoefficiens* USDA110 at 1×10^7^ cells per seedling. Plants were grown in a phytotron (Koito Industries) under 270‍ ‍μmol photons m^–2^ s^–1^ of photosynthetically active radiation (400–700‍ ‍nm) with a 16-h light/8-h dark cycle at 25/20°C for 30 days. A nitrogen-free sterilized nutrient solution was periodically supplied to the pots (Inaba *et al.*, 2012).

Thirty days after the inoculation, a soil suspension (30‍ ‍mL) prepared from 10‍ ‍g of fresh soil from the Kashimadai Experimental Station (Tohoku University: 38°27″37′N, 141°5″33′E) was added to the vermiculite in the pot after the aboveground plant parts were harvested as previously described (Fig. 2) (Inaba *et al.*, 2012). The pots were incubated in the phytotron for an additional 15 days. Nitrate or nitrite (50‍ ‍mL of 5‍ ‍mM) was then applied to the pot before a further 3-h incubation until N_2_O gas sampling and fungal isolation (Fig. 2).

### Analysis of N_2_O isotopomers

N_2_O isotopomer ratios were measured on an isotope ratio mass spectrometer (IRMS) (Finnigan MAT252; Thermo Fischer Scientific). Head space gas in the pot of the model system was first sampled into an evacuated 20-mL glass vial with a butyl rubber septum. After transport to the Science Tokyo Laboratory, an aliquot (0.5–10‍ ‍mL) of the gas sample was diluted with ultra-pure nitrogen gas to prepare a 0.3–10 ppm N_2_O/N_2_ mixture in a 100-mL glass container equipped with a vacuum stopcock. The diluted sample was then introduced into an online system consisting of a vacuum line, concentration traps, chemical traps for removing CO_2_ and H_2_O, a gas chromatograph (HP6890; Agilent Technologies), and IRMS (Toyoda *et al.*, 2005). Isotopomer ratios were assessed by a mass ana­lysis of mole­cular (N_2_O^+^) and fragment (NO^+^) ions of N_2_O for both the sample and reference gas. The notation of isotopomer ratios is shown below and the calibration procedure has been described in detail elsewhere (Toyoda and Yoshida, 1999). The typical precision of the ana­lysis was better than 0.1‰ for δ^15^N^bulk^ and better than 0.5‰ for δ^15^N^α^ and δ^15^N^β^.

*δ*^15^N*^i^*=^15^*R^i^*_sample_/^15^*R^i^*_std_–1, where *i*=α, β, or bulk (1)

*SP*=*δ*^15^N^α^–^15^N^β^ (2)

In equation (1), ^15^*R*^α^ and ^15^*R*^β^ represent the ^15^N/^14^N ratios at the center and terminal sites of nitrogen atoms, respectively; ^15^*R*^bulk^ indicates the average isotope ratios for ^15^N/^14^N (=(*δ*^15^N^α^+*δ*^15^N^β^)/2). The subscripts “sample” and “std” indicate the isotope ratios for the sample and the standard (atmospheric N_2_), respectively. In practice, laboratory reference N_2_O gas, the *δ*^15^N*^i^* of which had been calibrated against atmospheric N_2_ by the conversion of NH_4_NO_3_ with known *δ*^15^N_NH4_ and *δ*^15^N_NO3_ values to N_2_O, was measured with the sample.

### Fungal isolation and cultivation

Degraded nodules from the model pot system (45 days after sowing) were crushed with a glass rod in a Petri dish and suspended in distilled water. Organisms were observed under the phase constant mode (BX51 and DP71 CCD camera, Olympus). Single spores from the macerates of root nodules were isolated on agar plates consisting of Corn Meal Agar (CMA) medium (Nissui) by a Skerman micromanipulator (Skerman, 1968). CMA medium had the following composition (in g L^–1^): Cornmeal extract, 2.0; agar, 15.0. Single spores were dragged on fresh agar plates (CMA) using the microhook of the Skerman micromanipulator to eliminate attaching bacterial cells on the spore under the microscope, and the agar segment containing a single spore was cut out and transferred to the center of a new CMA plate. The germination of hyphae from the isolated spore was confirmed by microscopy. After plates had been incubated at 25°C for one week, fungal isolates were obtained from mycelia around the front of the colony.

### Elucidation of the internal transcribed spacer (ITS) sequence

The genomic DNA of fungal isolates was prepared with an FP DNA SPIN Kit (MP Bio). ITS regions were amplified with two primer sets: 2234C/3126T (Ranjard *et al.*, 2001) and ITS1-F/ITS4-A (Torzilli *et al.*, 2006). Cycling conditions were as follows: initial denaturation at 94°C for 2‍ ‍min, followed by 30 cycles at 94°C for 1‍ ‍min, at 60°C for 1‍ ‍min, and at 72°C for 2‍ ‍min, with a final extension at 72°C for 10‍ ‍min. Amplification reactions were performed in a 12.5-μL volume containing 1× Taq buffer, 0.2‍ ‍mM of the dNTP mixture, 1‍ ‍μM of each primer, 10‍ ‍ng of template DNA, and 0.25‍ ‍U of Ex Taq DNA polymerase (Takara). The PCR products of amplified nuclear sequences were ligated into the pTAC-1 Vector with the DynaExpress TA PCR Cloning Kit (BioDynamics Laboratory), transformed into *Escherichia coli* DH5α cells with the competent high *E. coli* DH5 α Kit (TOYOBO) and plated according to the kit protocols. Colonies identified through blue/white screening were cultured in Luria-Bertani broth with 100‍ ‍μg mL^–1^ ampicillin at 37°C for 1 day. Sanger sequencing ana­lyses of the ITS region were conducted by Nihon Gene Research Laboratories. A phylogenetic ana­lysis of fungal ITS sequences was conducted as previously described (Watanabe *et al.*, 2011).

### Nitrogen transformation by fungal isolates

Each fungal strain was precultured at 25°C on a rotary shaker (120‍ ‍rpm) in a test tube containing glycerol-peptone broth medium (GP medium). GP medium had the following composition (in g L^–1^): Bacto peptone, 2.0 (Thermo Fischer Scientific); glycerol, 30; KH_2_PO_4_, 1.36; MgSO_4_·7H_2_O, 0.2; NaNO_2_, 0.069; NaNO_3_, 0.085; FeSO_4_·7H_2_O, 0.1; FeCl_3_·6H_2_O, 0.1; ZnCl_2_, 0.02; CuSO_4_·5H_2_O, 0.01; NaMoO_4_·2H_2_O, 0.005; CoCl_2_·6H_2_O, 0.002; MnCl_2_·4H_2_O, 0.0016; H_3_BO_4_, 0.001; and citric acid, 0.1. The medium was adjusted with KOH to pH 7.5. After 3 days, 1‍ ‍mL of the culture was transferred to 19‍ ‍mL of fresh GP medium with and without 1‍ ‍mM nitrite or 1‍ ‍mM nitrate in a 120-mL vial. After the inoculation, the flask was sealed and then incubated at 25°C on a rotary shaker (120‍ ‍rpm).

Aliquots of the head space gas and culture were analyzed after 3, 5, and 7 days of the incubation as described below. N_2_O concentrations under nitrate and nitrite conditions were measured using gas chromatographs (GC14BpsE and GC2014; Shimadzu) equipped with electron capture and thermal conductivity detectors, respectively. O_2_ concentrations were measured using a gas chromatograph (GC2014; Shimadzu) equipped with a thermal conductivity detector. Nitrite (Keeney and Nelson, 1982) or nitrate (Cataldo *et‍ ‍al.*, 1975) in the medium was measured colorimetrically (Sameshima-Saito *et al.*, 2006), and the dry weight of cells was used as an indicator of cell growth.

### ^15^N-tracer experiment

The fungal isolates FK2 and FK7 were pre-cultured at 25°C on a rotary shaker (120‍ ‍rpm) in GP medium. After 3 days, 0.12‍ ‍mL of the culture was transferred to 2.18‍ ‍mL of fresh medium containing 1‍ ‍mM ^15^N-labeled nitrite (99 atom%; sodium salts; Cambridge Isotope Laboratories) in a 14-mL vial. After the inoculation, the flask was sealed and incubated at 25°C on a rotary shaker (120‍ ‍rpm). The head space gas was analyzed by gas chromatography-mass spectrometry (GC-MS) (GCMS-QP2010 Plus; Shimadzu) (Isobe *et al.*, 2011) equipped with a CP-PoraBOND Q (50 m×0.32‍ ‍mm) capillary column (Agilent Technologies) 5 days after the incubation.

### Microbial community ana­lysis

Soybean plants (cv. Enrei) were harvested on September 9, 2010 (99 days after sowing) in the Kashimadai Experimental Station. After soybean roots were washed well with tap water, nodules were manually collected from the root systems. Nodules were sampled from the model pot systems (45 days after sowing) in a similar manner. The fungal isolates FK1, FK2, and FK3 were cultured with CMA medium at 25°C for 3 days.

Microbial DNA was extracted from cultured cells (FK1, FK2, and FK3) and the surface of nodules using a Fast DNA SPIN Kit for soil (Qbiogene). The nodule sample (0.5 g) was suspended in DNA extraction solution in a 2-mL screw-capped tube. After the addition of glass beads, the tube was processed in a bead beater (Mikro-dismembrator S; B. Braun Biotech International) at 2,600×*g* for 1‍ ‍min. A ribosomal intergenic spacer ana­lysis (RISA) was performed to evaluate microbial DNA using the fungal primer sets of 1406f/3126T as previously described (Inaba *et al.*, 2009)

### Nucleotide sequence accession numbers

Nucleotide sequences of the ITS obtained from fungal isolates in the present study have been deposited in the DDBJ/EMBL/GenBank database under accession numbers LC034156 to LC034167.

## Results

Table 1 shows the SP values and concentrations of N_2_O under the three experimental conditions. When soybeans were inoculated with USDA110Δ*nosZ* and supplied with nitrate (experiment A), the resulting SP value and N_2_O concentration were –3.6±2.7‰ (1s, *n*=4) and 47±22 ppm, respectively; however, the replacement of the N source from‍ ‍nitrate to nitrite (experiment B) significantly increased‍ ‍both the SP value (4.2±0.5‰, *n*=3) and N_2_O concentration (103±11 ppm) (*P*<0.05, Table 1). Moreover, when the bradyrhizobial inoculant was replaced with USDA110Δ*nirK*Δ*nosZ* incubated with nitrite (experiment C), the SP value further increased (to 14.4±2.8‰, *n*=4) (*P*<0.05, Table 1), whereas the N_2_O concentration decreased to a level similar to that in experiment A.

**Fig. 1.**
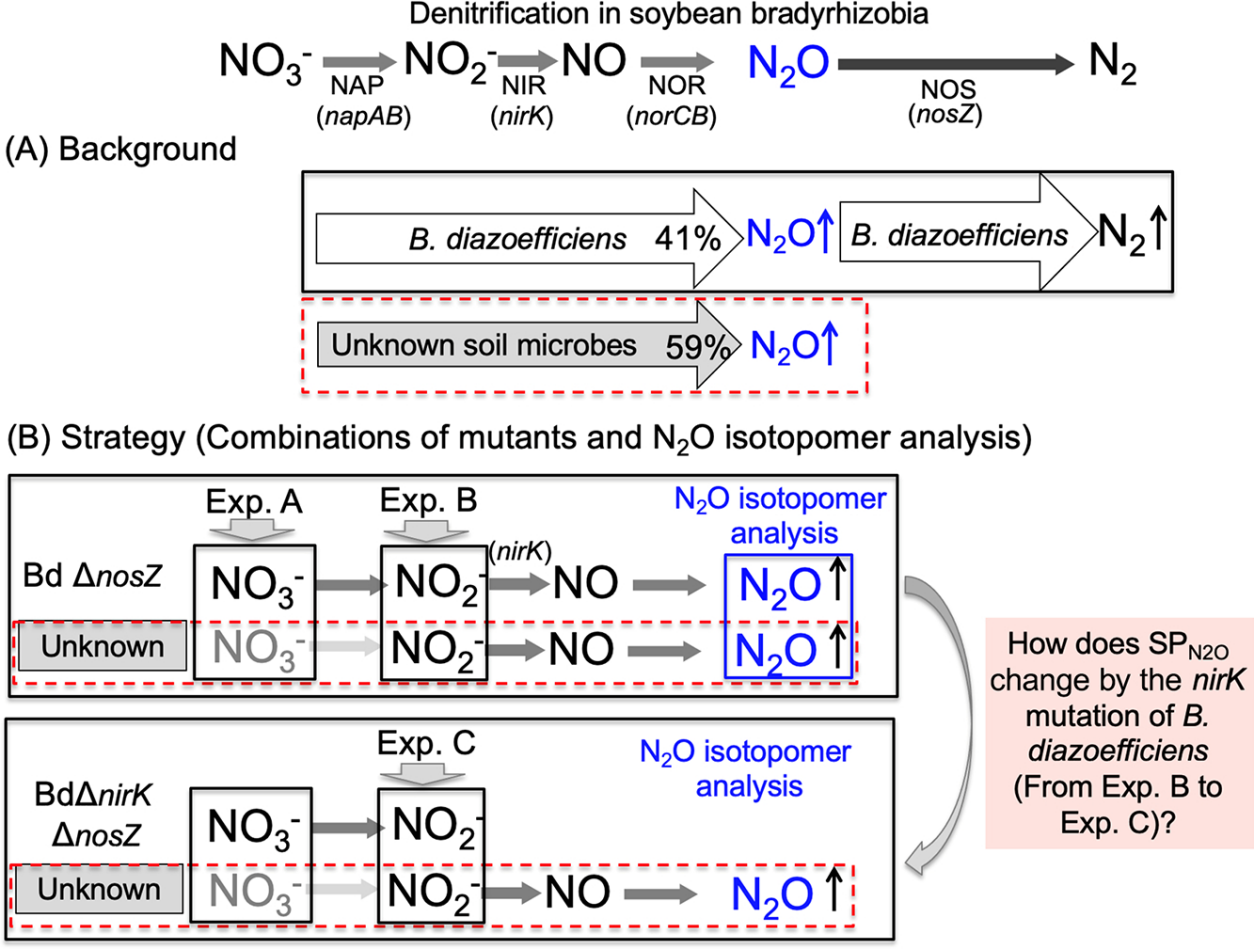
Schematic presentation of the background and strategy to elucidate pathways of N_2_O emissions from degraded nodules in a model pot system. (A) Summary of a previous experiment (Inaba *et al.*, 2012). (B) Outline of our strategy to identify unknown soil microbes (Unknown). BdΔ*nosZ* and BdΔ*nirK*Δ*nosZ* indicate the *nosZ* and* nirK/nosZ* mutants of *Bradyrhizobium diazoefficiens* USDA110, respectively (Inaba *et al.*, 2012). Experiments A, B, and C and the corresponding arrowheads show the addition of inorganic nitrogen and the *B. diazoefficiens* mutants used (Fig. 2 and Table 1).

**Fig. 2.**
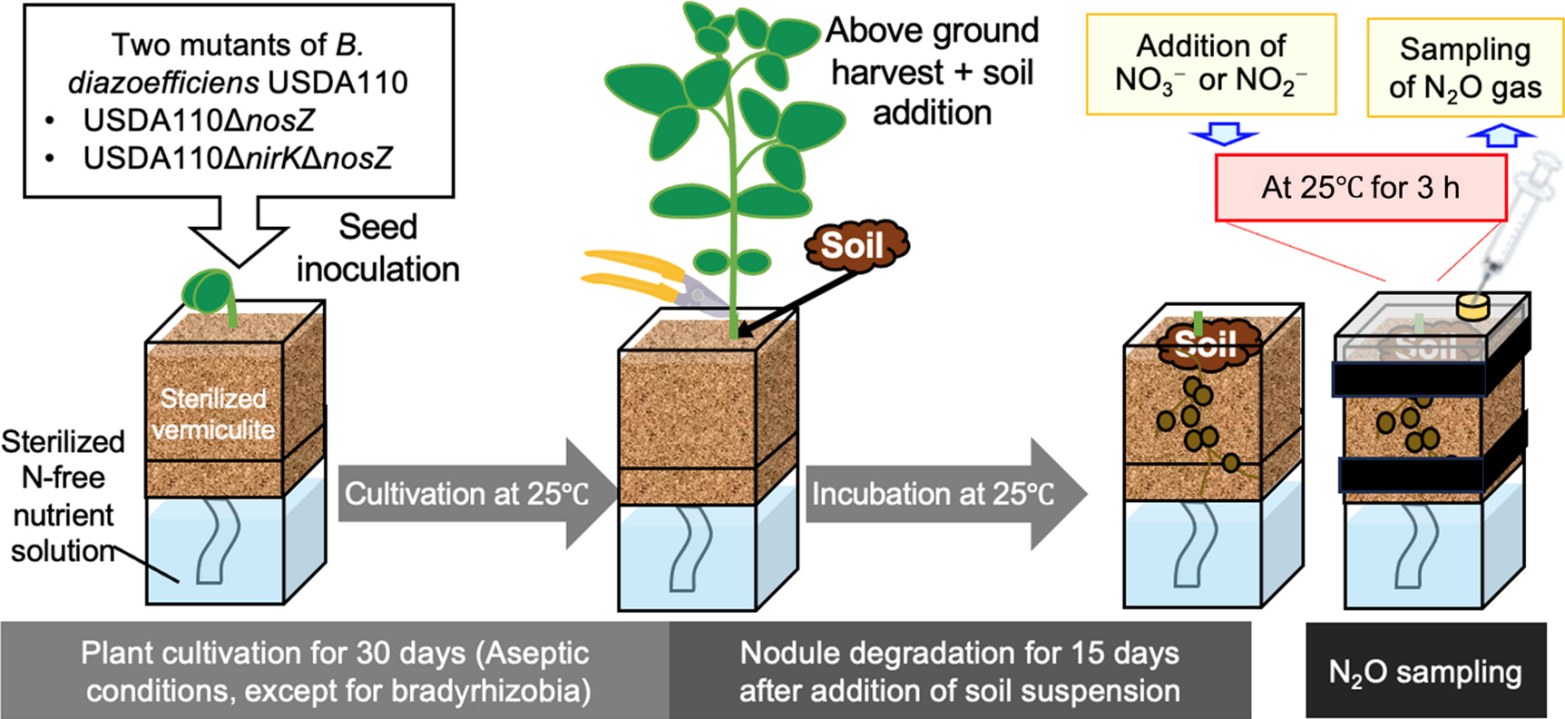
Model pot system to sample N_2_O from degraded nodules in the soybean rhizosphere with one of two mutants of *Bradyrhizobium diazoefficiens* USDA110.

Since USDA110Δ*nirK*Δ*nosZ* was unable to produce N_2_O from nitrite (Fig. 1B), unknown soil microbes may have largely decided the SP value of N_2_O in experiment C (Table 1). Fungal denitrification has been shown to emit N_2_O physiologically via cytochrome P450*nor* (Shoun *et al.*, 2012), which differs from bacterial NO reductase encoded by the *norBC* genes (Kuypers *et al.*, 2018). The N_2_O isotopomer ana­lysis is a useful tool for discriminating between fungal and bacterial denitrification processes that emit N_2_O (Yoshida and Toyoda, 2000). The site preference values of N_2_O (SP_N2O_) of fungi (approximately 16–37‰) are generally higher than those of bacteria (approximately 0‰) (Maeda *et al.*, 2015). Since the mutation of the *nirK* gene (encoding dissimilatory nitrite reductase) in symbiotic bradyrhizobia significantly increased SP_N2O_ from 4.2‰ to 14.4‰ in the presence of nitrite (Table 1, experiments B and C, Fig. 1), these results suggest that N_2_O emissions via fungal denitrification occurred in the model pot system of the soybean rhizosphere (Fig. 2).

**Fig. 3.**
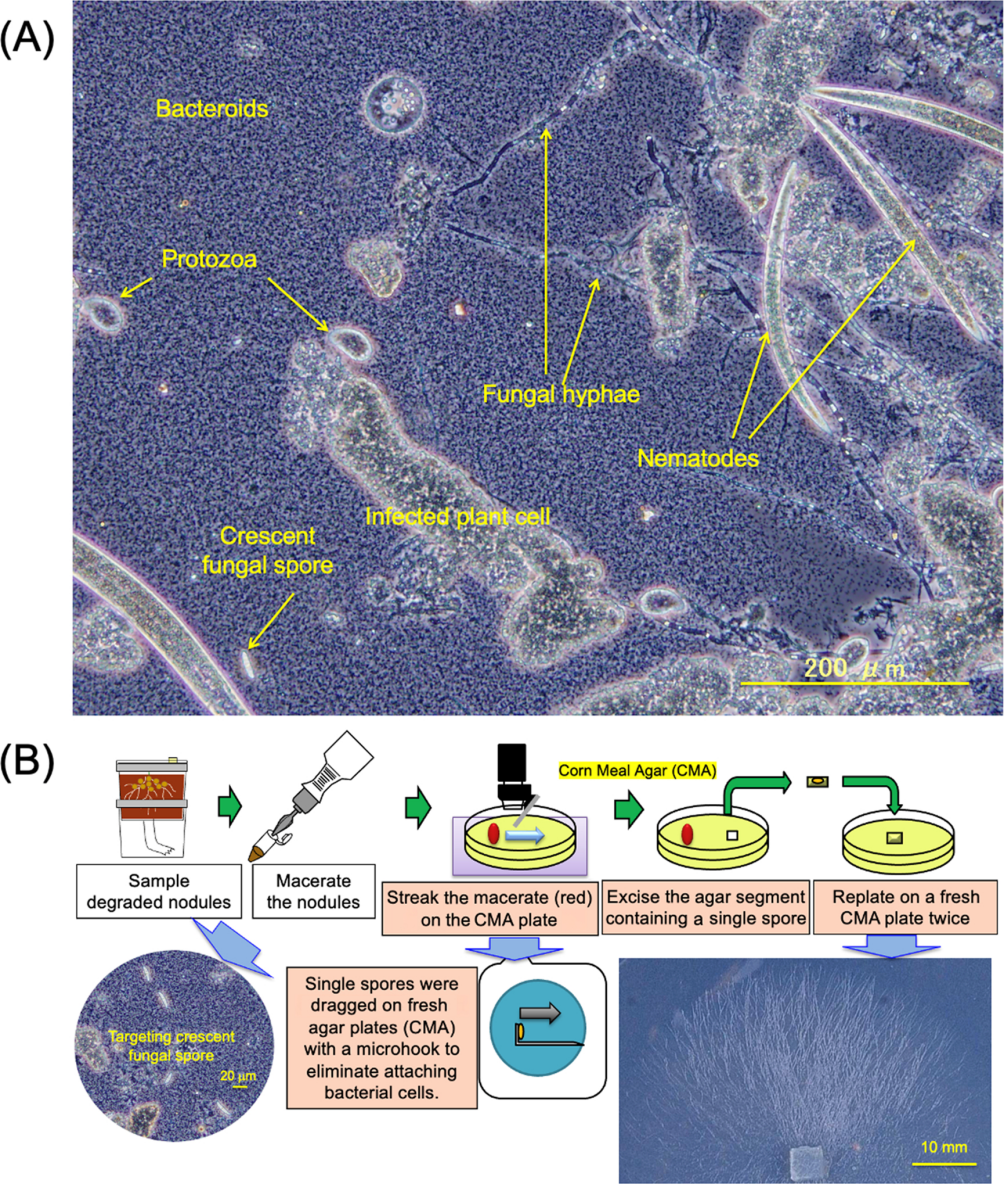
Microscopic observations of N_2_O-emitting nodules (A) and the isolation of single spores (B). Bacteroids of soybean bradyrhizobia are very small, abundant bacterial cells in panel A.

**Fig. 4.**
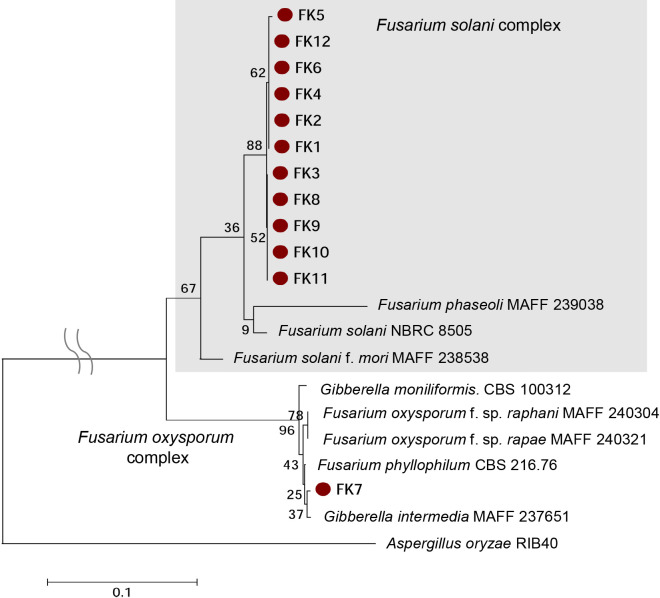
Phylogenetic tree of isolated fungi based on internal transcribed spacer sequences between 18S and 28S ribosomal RNA genes.

When we microscopically observed the degraded nodules emitting N_2_O from the model pot system (Fig. 2), we identified fungal hyphae and crescent spores as well as nematoda, protozoa, and very small bradyrhizobial cells (Fig. 3A). We then attempted to microscopically isolate single spores and cultivate them on agar plates (Fig. 3B). We ultimately obtained 12 fungal isolates (FK1 to FK12) and phylogenetically identified them as *Fusarium* species based on the DNA sequences of their ITS sequences (Fig. 4); most isolates were classified in the *Fusarium solani* complex.

**Fig. 5.**
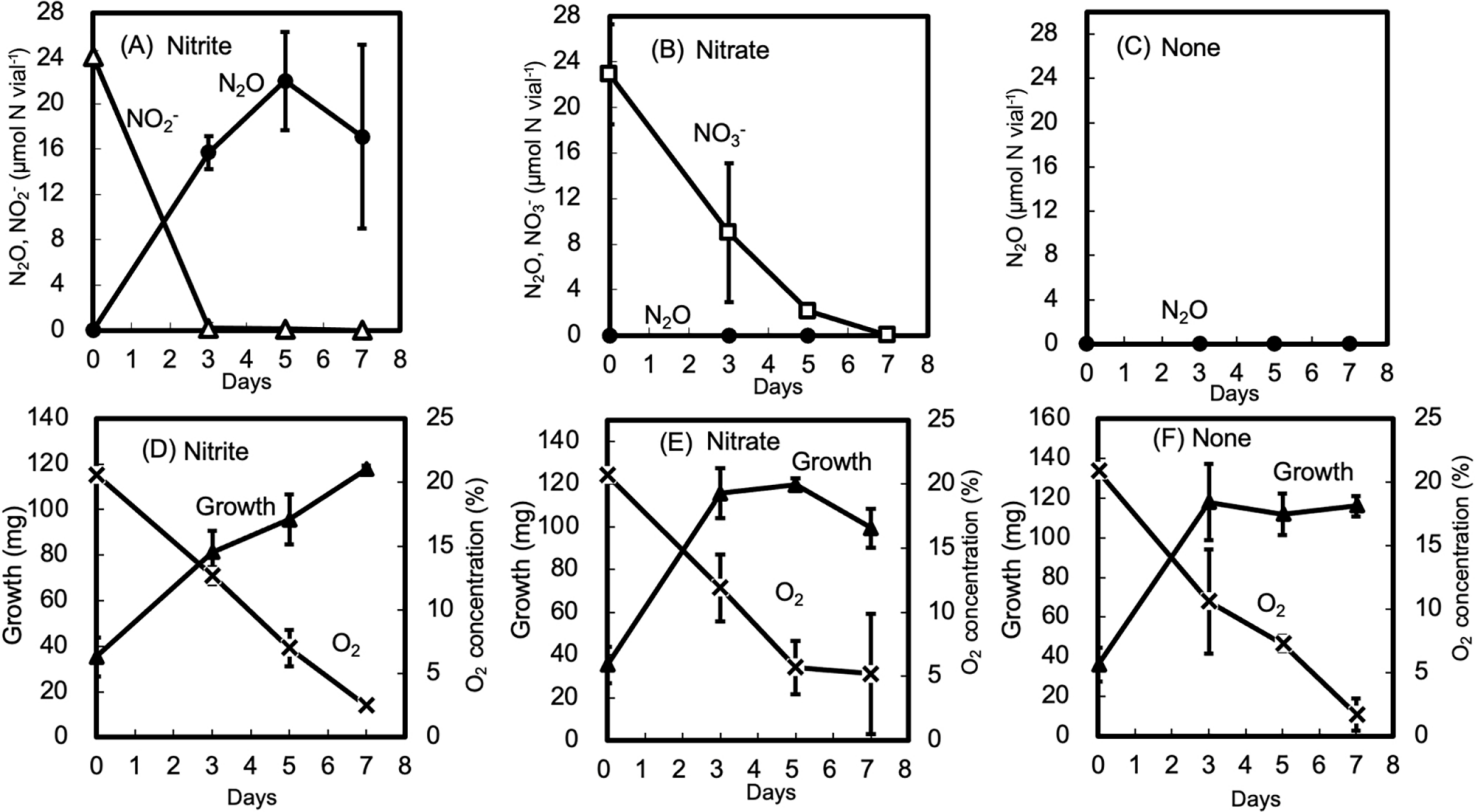
N_2_O emissions and growth of the *Fusarium* FK2 culture in the presence of nitrite (A, D) or nitrate (B, E). “None” (C, F) indicates no addition of nitrite or nitrate. Symbols: N_2_O (●); nitrite (△); nitrate (□); cell growth (▲); O_2_ concentration (×, [v/v %]).

When the fungal isolate FK2 was cultivated in GP medium, N_2_O was emitted from FK2 in medium supplemented with nitrite (Fig. 5A), but not that supplemented with nitrate (Fig. 5B) or none (no addition of nitrite or nitrate) (Fig. 5C). Over time, O_2_ was consumed, and fungal growth was observed in GP media irrespective of the treatments (Fig. 5D, E, and F).

Table 2 summarizes N_2_O emissions from the 12 isolates grown in the same sets of media and culture conditions as FK2 (Fig. 5). All isolates, except for FK1, strongly converted nitrite to N_2_O, whereas N_2_O emissions were not observed in any isolate in the presence of nitrate (Table 2).

To investigate whether N_2_O was emitted by *Fusarium* isolates with nitrite, a tracer experiment was conducted in GP media supplemented with ^15^N-labeled nitrite (99 atom%) (Table 3) using the isolates FK2 and FK7 representing the *Fusarium solani and oxysporum* complexes (Fig. 4). The results obtained clearly show that ^15^N^15^NO (*m/z*=46) was the dominant chemical species of N_2_O molecules in the gas phases of the FK2 and FK7 cultures (Table 3), suggesting that N_2_O was produced by these fungi exclusively from nitrite in accordance with the following equation: 2NO_2_^–^+6H^+^+4e^–^→N_2_O+3H_2_O.

**Fig. 6.**
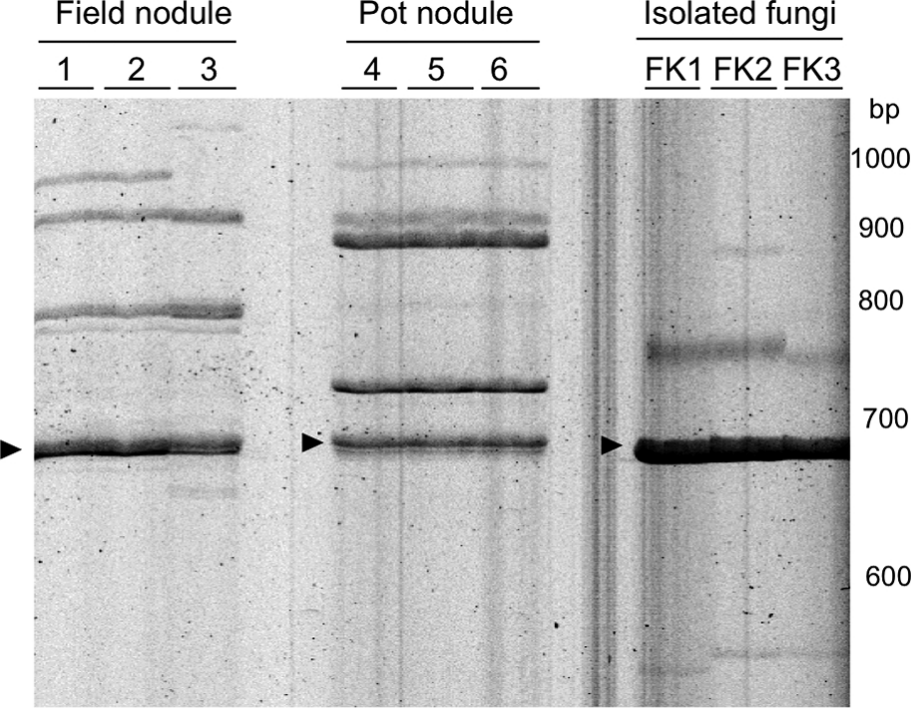
Comparison of ribosomal intergenic spacer ana­lysis profiles of degraded soybean nodules in field and pot experiments with those of isolated fungi (FK1, FK2, and FK3). The arrowhead indicates the common signal band that was likely shared by the field and pot nodules and isolated *Fusarium* fungi. Numbers indicate individual replicates in field nodules (1, 2, and 3) or the model pot system (4, 5, and 6).

To establish whether our *Fusarium* isolates existed in N_2_O-emitting soybean nodules in (i) the rhizosphere of field-grown soybeans and (ii) our model pot system (Fig. 2), we compared their fungal RISA profiles with those of our isolated fungi (Fig. 6). A common band was shared by nodules in the field/pot and isolated *Fusarium* fungi (Arrowheads in Fig. 6). These results suggest that our *Fusarium* isolates exist in soybean fields and may contribute to N_2_O emissions from the soybean rhizosphere at field and pot levels.

## Discussion

Fungi have not been regarded as relevant to the microbial nitrogen-cycling network in the plant rhizosphere (Baggs, 2011; Kuypers *et al.*, 2018), although ectomycorrhizal fungi associated with plants improve nitrogen absorption and transport via their mycelia (Meena *et al.*, 2023). *Fusarium* species are cosmopolitan soil fungi belonging to Ascomycota, and often cause significant economic losses of crops as phytopathogens (Todorović *et al.*, 2023). In a previous study, a *Fusarium* sp. was frequently detected in the N_2_O-emitting soybean rhizosphere using a community ana­lysis (Inaba *et al.*, 2009). *F. oxysporum* and *F. solani* have been shown to denitrify to N_2_O (Shoun and Tanimoto, 1991). In addition, fungi often account for a large percentage of the soil biomass and have a large N_2_O production potential (Maeda *et al.*, 2015).

**Fig. 7.**
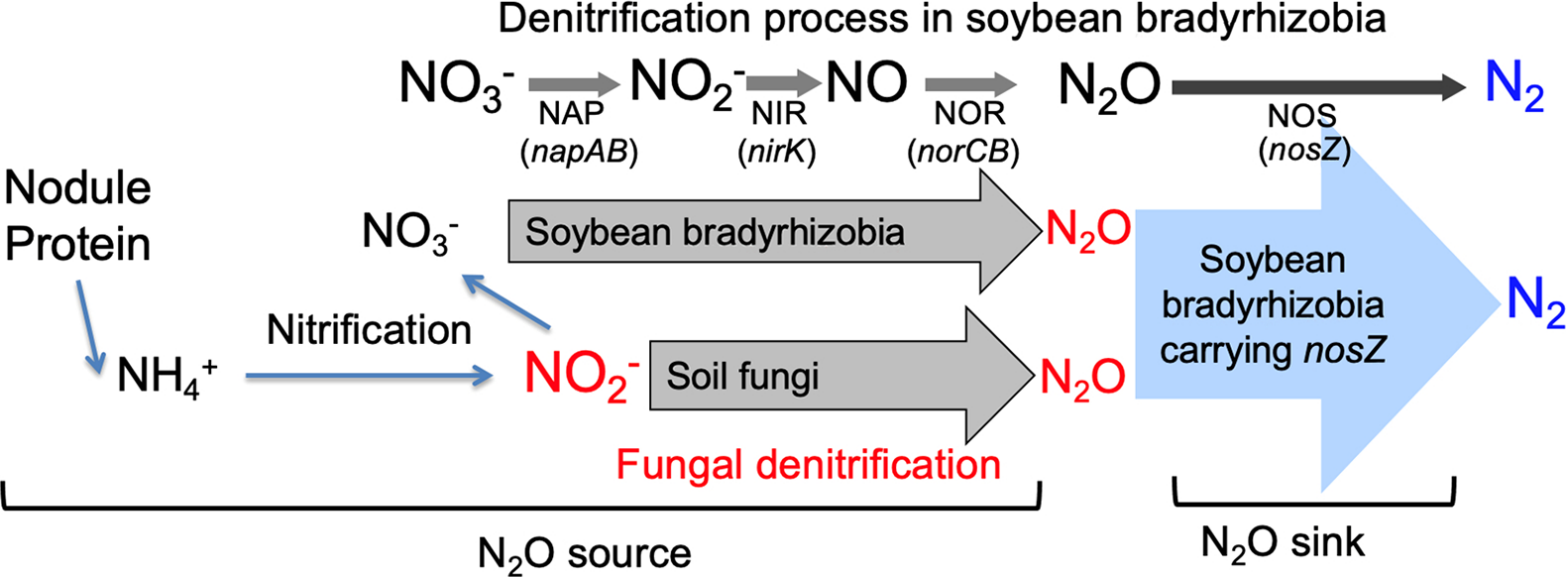
Schematic presentation of the nitrogen cycle in the soybean nodule rhizosphere.

In the present study, the combination of the N_2_O isotopomer ana­lysis and use of bradyrhizobial mutants in our model pot system suggested the presence of fungal N_2_O emissions in the soybean rhizosphere. Subsequent fungal isolation, nitrogen transformation assays, and the community ana­lysis indicated that *Fusarium* fungi mediated N_2_O emission processes from nitrite in the soybean rhizosphere (Fig. 7). Although the process of nitrite supply has not yet been confirmed experimentally, it is possible that the nitrite supply is relevant to nitrification in the rhizosphere (Sánchez and Minamisawa, 2019).

Since this was the first study to adopt the N_2_O isotopomer ana­lysis of plant rhizosphere systems, we would like to fully discuss the implications of the results obtained. Since the SP value obtained (–3.2‰) in experiment A (nitrate as the sole inorganic nitrogen source; Table 1) was within the range of the values reported for N_2_O produced by bacterial denitrification (–6 to 0‰; Toyoda *et al.*, 2017), we considered N_2_O produced from nitrate to be solely attributed to bacterial denitrification, while N_2_O reduction by microbes introduced from the soil suspension added was negligible. N_2_O produced in experiment B (nitrite as the sole inorganic nitrogen source; Table 1) had higher SP values (4.2‰). Three microbial processes are known to produce N_2_O with higher SP values than that by bacterial denitrification: bacterial and archaeal ammonia oxidation (13–37‰, Toyoda *et al.*, 2017) and fungal denitrification (16–37‰, Maeda *et al.*, 2015). Since ammonia was not added as a substrate in the present study, we considered N_2_O production by ammonia oxidation to be unlikely; however, the possibility of ammonia forming through the degradation of organic compounds in soybean nodules cannot be excluded. Therefore, we considered N_2_O to be produced from nitrite by fungi as well as denitrifying bacteria. If we assume that the amount of N_2_O produced by bacterial denitrification was nearly the same in experiments A and B, the SP value of N_2_O by fungal denitrification, SP_fungi_, is then calculated from the mass balance as follows:

*n*_B_=*n*_A_+*n*_fungi_ (3)

SP_B_×*n*_B_=SP_A_×*n*_A_+SP_fungi_×*n*_fungi_ (4)

SP_fungi_=(SP_B_×*n*_B_–SP_A_×*n*_A_)/(*n*_B_–*n*_A_), (5)

where *n* is the amount of N_2_O produced and may be replaced with the N_2_O concentration, and *n*_A_ is assumed to be equal to the amount of N_2_O produced by bacterial denitrification (*n*_bacteria_). The SP_fungi_ value (10.7‰) obtained was lower than the lowest SP value reported for fungi (16‰; Maeda *et al.*, 2015), which indicates that *n*_bacteria_ in experiment B was larger than *n*_A_.

The result of experiment C may be explained by the suppression of bacterial denitrification due to the lack of *nirK* and a relative increase in the contribution from fungal denitrification. If we assume that SP_bacteria_=SP_A_ and SP_fungi_ is within the range of reported values, the contribution of N_2_O production from fungal denitrification, *x*, in experiments B and C is then calculated as follows:

SP*_i_*=(1–*x_i_*)×SP_A_+*x_i_*×SP_fungi_ (6)

*x_i_*=(SP*_i_*–SP_A_)/(SP_fungi_–SP_A_), (7)

where *i*=B or C.

With the lowest reported value of SP_fungi_ (16‰), *x*_B_ and *x*_C_ are calculated as 0.40 and 0.92, respectively, whereas with the highest value of SP_fungi_ (37‰), they are 0.19 and 0.44, respectively. The N_2_O concentrations observed in experiments A–C were in accordance with the former case (*n*_B_≅*n*_A_+*n*_C_, where A and C correspond to bacterial and fungal denitrification, respectively). Taken together with SP_fungi_ estimated by eq. (5), the SP value of N_2_O produced by fungi in the present study appeared to be close to the lower end of the reported range. Regardless of the SP_fungi_ value, our results indicate that N_2_O is produced by fungi in soybean nodules from nitrite, but not from nitrate, and also that the *nirK* mutation increased the fungal contribution to N_2_O production.

Several strategies have recently been proposed to mitigate N_2_O emissions by N_2_O-reducing microbes in agricultural settings (Bakken and Frostegård, 2020; Minamisawa, 2022; Hiis *et al.*, 2024). One approach is to develop inoculants for legumes that are strong N_2_O reducers (Itakura *et al.*, 2013). *Bradyrhizobium ottawaense* exhibited stronger N_2_O-reducing activity than *B. diazoefficiens*, and had a greater potential to efficiently mitigate N_2_O emissions from the soybean rhizosphere in fields (Wasai-Hara *et al.*, 2020, 2023). Another strategy is to grow N_2_O-reducing bacteria in digestates from biogas production and apply them to agricultural lands (Hiis *et al.*, 2024). These promising options warrant further study and validation.

The present study revealed the potential of *Fusarium* to emit N_2_O in the soybean rhizosphere, which needs to be tested by careful experimental designs in outdoor soybean fields in the future. Laughlin and Stevens (2002) found that the addition of cycloheximide as a fungal inhibitor to grassland soil reduced N_2_O emissions, suggesting that fungi were responsible for the majority of N_2_O produced. They proposed that fungal denitrification may be of ecological significance because N_2_O is the dominant gaseous end product (Laughlin and Stevens, 2002). If this is the case, antifungal organisms and agents may reduce N_2_O emissions from the soybean rhizosphere (Shen *et al.*, 2021). Our results on the N_2_O emission potential of fungi in the soybean rhizosphere suggest that specific inhibitors or biological competitors of *Fusarium* species will reduce N_2_O emissions from agricultural soils.

## Citation

Moriuchi, M., Kuzunuki, K., Ikenishi, F., Sameshima, R., Nakagiri, A., Toyoda, S., et al. (2025) *Fusarium* Fungi Produce Nitrous Oxide (N_2_O) from Nitrite (NO_2_^–^) in a Model Pot System Simulating the Soybean Rhizosphere. *Microbes Environ ***40**: ME24092.

https://doi.org/10.1264/jsme2.ME24092

## Figures and Tables

**Table 1.** Site preference (SP) and amount of N_2_O evolved from the rhizosphere of soybean plants inoculated with one of two mutants of wild-type *Bradyrhizobium diazoefficiens* USDA110: USDA110Δ*nosZ* or USDA110Δ*nirK*Δ*nosZ*^†^

Experiment	Experimental conditions		SP (‰)	N_2_O (ppm)
	Inoculant	Treatment		
A	USDA110Δ*nosZ*	Nitrate	–3.6±2.7*^a^*	47±22*^a^*
B	USDA110Δ*nosZ*	Nitrite	4.2±0.5*^b^*	103±11*^b^*
C	USDA110Δ*nirK*Δ*nosZ*	Nitrite	14.4±2.8*^c^*	47±26*^a^*

^†^ SP and the amount of N_2_O gas were assessed in three biological replicates after 30 days of plant cultivation followed by decapitation, soil addition, and 15 days of further incubation (Fig. 2). Nitrate or nitrite were added 3‍ ‍h before N_2_O sampling (Fig. 2). Values are expressed as the mean±standard deviation. Values marked with the same letter (*a–c*) within a column do not significantly differ according to Tukey’s test for pairwise mean comparisons at α=0.05.

**Table 2.** N_2_O production in the presence of nitrite or nitrate in cultures of fungal isolates from degraded nodules in the model pot system^†^

Isolates	N_2_O emission (N_2_O-N μmol vial^–1^)	
	Nitrite	Nitrate
FK1	ND	ND
FK2	17.7±2.3	ND
FK3	17.2±1.0	ND
FK4	17.7±1.4	ND
FK5	16.1±2.6	ND
FK6	18.0±1.9	ND
FK7	6.4±1.3	ND
FK8	17.6±1.7	ND
FK9	18.0±1.8	ND
FK10	17.8±1.7	ND
FK11	18.2±2.6	ND
FK12	17.8±1.7	ND

^†^ N_2_O gas in vials was assessed in three biological replicates. These cultures were sampled 5 days after inoculation. Values are expressed as the mean±standard deviation. ND, not detected (<0.18 N_2_O-N μmol vial^–1^).

**Table 3.** ^15^N-nitrite tracer ana­lysis of cultures of fungal isolates^†^

Chemical species	*m/z*	N_2_O accumulation (N_2_O-N μmol vial^–1^)	
		FK2	FK7
^15^N^15^NO	46	1.62±0.17	1.03±0.10
^15^N^14^NO and ^14^N^15^NO	45	0.02±0.00	0.02±0.00
^14^N^14^NO	44	ND	ND

^†^ The amounts of different isotopes in culture vials of the isolates FK2 and FK7 of *Fusarium* sp. were assessed 5 days after the inoculation in three biological replicates. Values are expressed as the mean±standard deviation. ND, not detected (<3×10^–3^ μmol vial^–1^).
